# A Density Functional Valence Bond Study on the Excited States

**DOI:** 10.3390/molecules30030489

**Published:** 2025-01-22

**Authors:** Xun Wu, Peikun Zheng, Tingzhen Chen, Chen Zhou, Peifeng Su, Wei Wu

**Affiliations:** State Key Laboratory of Physical Chemistry of Solid Surfaces, Fujian Provincial Key Laboratory of Theoretical and Computational Chemistry, Department of Chemistry, College of Chemistry and Chemical Engineering, Xiamen University, Xiamen 361005, China; wuxun@stu.xmu.edu.cn (X.W.); pkzheng@stu.xmu.edu.cn (P.Z.); 20520241152142@stu.xmu.edu.cn (T.C.); supi@xmu.edu.cn (P.S.)

**Keywords:** valence bond theory, excited states, density functional valence bond method, electronic correlation, state interaction

## Abstract

The accurate description of excited states is crucial for the development of electronic structure theory. In addition to determining excitation energies, strong state interactions arise when electronic states with the same symmetry are degenerate or nearly degenerate, often requiring a multi-state treatment. These strong correlation effects and state interactions can be effectively handled by the Hamiltonian matrix correction-based density functional valence bond (hc-DFVB) method, a multi-reference density functional theory capable of accurately describing electronic state interactions. In this paper, we explore the low-lying excited states of four isoelectronic systems (C_2_H, CN, CO^+^, BO) using valence bond methods, including the valence bond self-consistent field (VBSCF) and hc-DFVB methods. Our results show that the hc-DFVB method provides significantly better excitation energies compared to VBSCF. Furthermore, hc-DFVB can reliably predict the correct ordering of excited states, whereas VBSCF shows some ordering inconsistencies. By categorizing the VB structures into groups based on point group symmetry, we can extract the key structural contributions and bonding pictures of each state from the weight distribution of these groups. Additionally, we study the potential energy curves for lithium fluoride (LiF) and a mixed-valence spiro cation, demonstrating the superior performance of hc-DFVB when applied to the study of near-degenerate excited states in the avoided crossing region.

## 1. Introduction

The accurate description of electronic excited states is crucial in spectroscopy and photochemical reactions and remains a challenge in the development of electronic structure theory. Time-dependent density functional theory (TD-DFT) [1,2,3,4,5] has emerged as a widely used approach for studying excited-state properties, particularly for large systems, due to its moderate computational cost and user-friendly, black-box nature. However, many electronic excited states exhibit strong correlation effects, which require careful treatment of static correlation. For such states characterized by strong electronic correlations or multiple excitation features, TD-DFT often fails to provide reliable results. This limitation arises mainly because the inherently multiconfigurational nature of these states cannot be adequately represented by a single determinant.

In contrast, multi-configurational wave function theory (WFT) captures static correlation by expanding the wave function as a linear combination of several configuration state functions (CSFs) in the multi-configuration self-consistent field (MCSCF) method. The complete active space self-consistent field (CASSCF) method [6], whose wave function is constructed with all the CSFs in a given active space, is the most widely used MCSCF method based on molecular orbitals (MOs). To achieve more accurate treatment in MO theory, it is necessary to further cover dynamic correlation based on the MCSCF wave function and such treatment results in the post-MCSCF methods. The most commonly used post-MCSCF methods include the complete active space second-order perturbation theory (CASPT2) [7] and multi-reference configuration-interaction (MRCI) [8].

As an alternative to MO-based WFT theory, valence bond (VB) theory, which provides clear chemical pictures and deep insights into chemical bonds and reaction mechanisms, is also a multi-configurational method that captures static correlation. In the most fundamental valence bond self-consistent field (VBSCF) method [9,10], the wave function is expanded as a linear combination of VB structures, usually constructed using localized atomic orbitals or block-localized orbitals to retain the classical concept of Lewis structures. Based on the VBSCF wave function, the dynamic correlation can be further included in the post-VBSCF methods, including valence bond perturbation theory (VBPT2) [11], valence bond configuration interaction (VBCI) [12].

However, the accurate treatment of both static and dynamic correlation within WFT is computationally expensive, making it impractical even for moderate-sized systems. An alternative approach to address both static and dynamic correlation in strongly correlated excited states is the combination of MCSCF wave functions with DFT, as employed in multi-reference density functional theory (MRDFT). There are two central problems when combining the MCSCF wave function and the DFT functional. The first problem is the double-counting error, which means some correlations could be included twice in both the WFT and DFT functional contributions since there is no exact definition for static and dynamic correlation. The second problem is known as the symmetry dilemma, which means that with the present approximated functionals, either the wrong energy is obtained with the correct electron spin density or the correct energy is obtained with the wrong electron spin density. Since the 1990s, various MO-based MRDFT schemes have been proposed to deal with the problems addressed above [13,14,15,16,17,18,19,20,21,22,23,24,25,26,27,28,29,30,31]. Recently, a series of MRDFT methods based on nonorthogonal VB orbitals, namely density functional valence bond (DFVB) methods, have been proposed [32,33,34,35]. Overall, MRDFT methods provide an accurate and economical way to treat both the static and dynamic correlation for strongly correlated systems, including excited states.

There are many VB studies on molecular excited states with modern classical valence bond methods, such as the studies on the lowest excited states (^1^Δ_g_ and ^1^Σ_g_^+^) of O_2_ [36], the ^1^Δ and ^1^Σ^+^ excited states of NF [37], the V state of ethylene [38], and the lowest-lying singlet electronic states of O_3_ and SO_2_ [39]. All these studies showed that although VBSCF can sometimes give correct qualitative descriptions for the excited states, the inclusion of dynamic correlation is still required to give a quantitatively accurate description of the excited states. Furthermore, when electronic states with the same symmetry are degenerate or near-degenerate, they tend to interact strongly with each other to form a conical intersection. A multi-state (MS) treatment is usually required to describe the correct topography of adiabatic potential energy curves (PECs) at conical intersections [40]. Since most trajectories do not precisely pass through the conical intersection seam, the PECs near the conical intersection region are avoided along the path and are therefore known as avoided crossings. In the MS treatment, the state interaction is considered by the construction of an effective Hamiltonian and the adiabatic states are obtained by the diagonalization of the effective Hamiltonian at the last step. The most widely used MS methods include multi-state CASPT2 (MS-CASPT2) [41], multi-configuration quasi-degenerate perturbation theory (MC-QDPT) [42], and their extended formulations, namely XMS-CASPT2 [43] and XMC-QDPT [44]. Recently Truhlar et al. have developed a series of MS methods based on multiconfiguration pair-density functional theory [45,46,47].

For the reasons explained above, the study of excited states requires consideration of both static and dynamic correlations, as well as electronic state interactions, within the selected quantum chemical methods. The Hamiltonian matrix correction based density functional valence bond (hc-DFVB) method [33] is one such approach. In hc-DFVB, the static correlation is considered with the VBSCF wave function and the dynamic correlation is considered with the DFT functional correction to each VB determinant. Since the last step of hc-DFVB is a diagonalization of the effective Hamiltonian, hc-DFVB is an MS method, making it suitable for studying systems with strong state interactions.

This paper aims to investigate the performance of the hc-DFVB method in studying systems that exhibit strong multi-reference characteristics and possess highly correlated excited states. The paper is organized as follows: first, a brief review of the hc-DFVB method is presented, followed by the computational details. Then, the low-lying excited states of four doublet radicals, namely C_2_H, CN, BO, and CO^+^, are explored by VBSCF and hc-DFVB and a scheme of VB structure classification based on point group symmetry is used to analyze the variation of weights of VB structures between different excited states. Finally, we also study the PECs for LiF and a mixed-valence spiro cation, 2,2′,6,6′-tetrahydro-4H,4′H-5,5′-spirobi[cyclopenta[*c*]pyrrole] molecule, (denoted as “spiro cation” in the following) to show the performance of hc-DFVB when applied to the study of near-degenerate excited states in the avoided crossing region.

## 2. Results and Discussion

### 2.1. Low-Lying Excited States of Doublet Radicals

#### 2.1.1. Construction of VB Structures

The point group of the four isoelectronic doublet molecules (C_2_H, CN, BO, CO^+^) is all C_∞*v*_ and its subgroup C_2v_ is applied in this section to determine the electronic states of the tested molecules. When the orientation of the molecules is aligned along the *z* axis, the Σ^+^ and Σ^−^ irreducible representations in the *C*_∞*v*_ point group correspond to A_1_ and A_2_ in the C_2v_ point group, respectively. While the degenerate Π_1_ and Π_2_ states in the *C*_∞*v*_ point group correspond to B_1_ and B_2_ in the C_2v_ point group, respectively. Especially, for the C_2_H system, the active orbitals are either localized on one C atom or localized on the remaining C-H fragment. Therefore, we can use “AB” to denote all four systems; here B can be an atom or a fragment (C-H).

**Figure 1:**
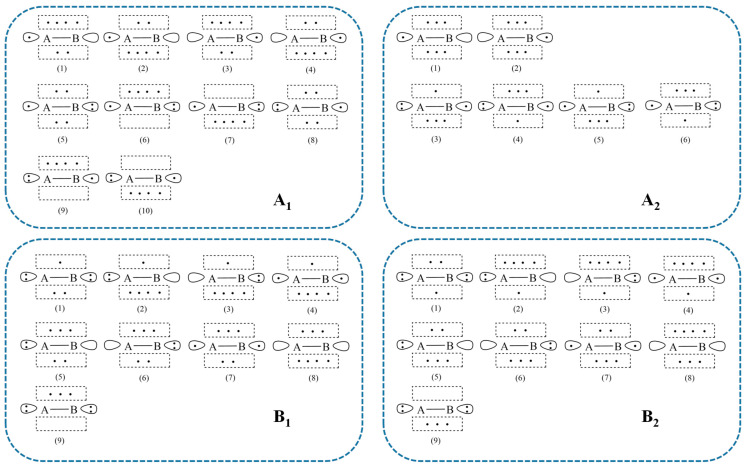
The category of VB structures according to the reduced point group of C_2v_ for C_2_H, CN, BO, and CO^+^.

To further analyze the variation of weights of VB structures in different electronic states, we classify the full VB structures in the active space into different categories according to their symmetries in the C_2v_ point group. The grouped VB structures for C_2_H, CN, BO, and CO^+^ are shown in Figure 1 and there are in total four sets of grouped VB structures, namely A_1_, A_2_, B_1_, and B_2_ sets:

(a) A_1_ set: because the total number of electrons of all the tested systems is odd, the A_1_ symmetry requires that the number of electrons occupied on orbitals in the *z* direction is also odd. There are two possible values (1 or 3) of the electrons occupied on the orbitals in the *z* direction. By considering all the distribution ways of the electrons in each direction, we can obtain a total of 10 groups of VB structures in this A_1_ set, namely (1) to (4) groups in which only one electron in the *z* direction and (5) to (10) groups in which three electrons in the *z* direction.
(b) A_2_ set: there is no orbital with the *a*_2_ irreducible representation included in the active space. However, according to the product rules of the C_2v_ point group, the product of *b*_1_ and *b*_2_ can yield the *a*_2_ symmetry. Therefore, the A_2_ symmetry requires that the number of electrons occupied on the orbitals along the *x*, *y*, and *z* directions are all odd. Similarly, we classify the VB structures in this A_2_ set into two categories according to the number of electrons in the *z* direction, namely groups (1) and (2) with one electron in the *z* direction and groups (3) to (6) with three electrons in the *z* direction.
(c) B_1_ set: in this case, the B_1_ symmetry requires that the number of electrons in the *x* direction is odd, while the numbers of electrons in the *y* and *z* directions are both even. There are in total nine groups, namely groups (1) to (4) with one electron in the *x* direction and groups (5) to (9) with three electrons in the *x* direction.
(d) B_2_ set: the B_2_ symmetry requires that the number of electrons in the *y* direction is odd, while the numbers of the electrons in the *x* and *z* directions are both even. There are a total of nine groups, namely groups (1) to (4) with one electron in the *y* direction and groups (5) to (9) with three electrons in the *y* direction.

For simplicity, we will denote the group (*i*) in the *J* set (*i* = 1–10; *J* = A_1_, A_2_, B_1_, B_2_) as *J*-G*i* in the following. For instance, the group (5) in B_1_ set is written as B_1_-G5.

#### 2.1.2. Vertical Excitation Energies

Table 1, Table 2, Table 3 and Table 4 show the deviations of vertical excitation energies calculated by MS-CASPT2, VBSCF, and hc-DFVB methods compared with the MRCISD+Q results. For all the tested systems, MS-CASPT2 performs extremely well with a mean unsigned deviation (MUD) within 0.1 eV except for C_2_H with a slightly higher deviation of 0.17 eV. The excitation energies calculated by hc-DFVB between B3LYP and BLYP functionals are very close to each other, i.e., the differences are usually within 0.1 eV. In contrast, the differences between B3LYP and PBE functionals increase to around 0.2 eV.

The correct prediction of the ordering of each state is fundamental and important in the study of molecular excited states. For the four tested systems, the VBSCF method can produce the correct ordering except for the 1^2^Σ^−^ and 2^2^Π states of C_2_H, i.e., the energetic ordering of these two states predicted by VBSCF is reversed. In contrast, the correct ordering is always obtained by hc-DFVB and MS-CASPT2.

It is encouraging that the hc-DFVB results are significantly improved compared to VBSCF for all the systems. For the C_2_H system, the MUD decreases from 1.04 eV for VBSCF to 0.82 eV for hc-DFVB with B3LYP functional. The decrease of MUD is also found for the CN, BO, and CO^+^ systems, where their MUDs decrease, respectively, from 0.44, 0.31, and 0.30 eV for VBSCF to 0.29, 0.19, and 0.09 eV for hc-DFVB with B3LYP functionals. The improvements of the hc-DFVB method over VBSCF demonstrate the importance of taking into account the dynamic electron correlation in the VB calculation.

Furthermore, we can find that the large errors of the excitation energies for VBSCF usually come from the high-lying excited states, e.g., VBSCF overestimates the excitation energies of the 2^2^Π states of C_2_H, CN, BO, CO^+^ by 1.10, 0.65, 0.73, and 0.39 eV, respectively. By including dynamic correlation, the hc-DFVB method provides improved accuracy for these high-lying states, except that large errors still exist for the C_2_H case, which can be attributed to the existence of some Rydberg characters for this excited state.

#### 2.1.3. The VB Structure Weights Gathered by Groups

**Figure 2:**
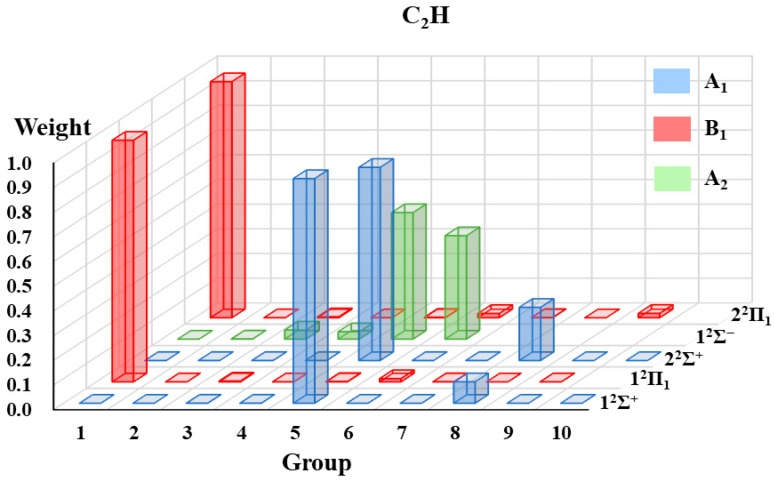
The weights of grouped VB structures for each state of C_2_H.

**Figure 3:**
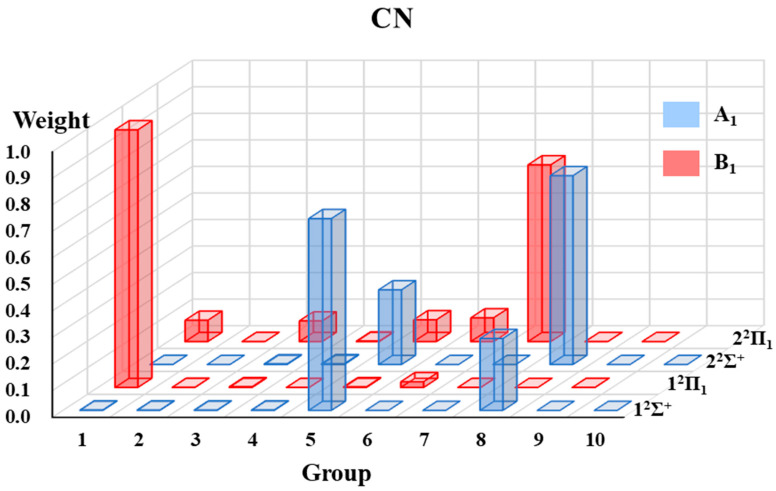
The weights of grouped VB structures for each state of CN.

**Figure 4:**
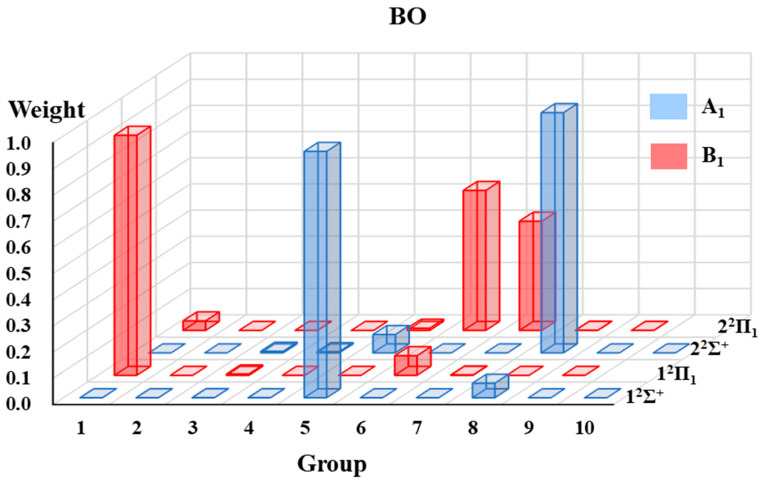
The weights of grouped VB structures for each state of BO.

**Figure 5:**
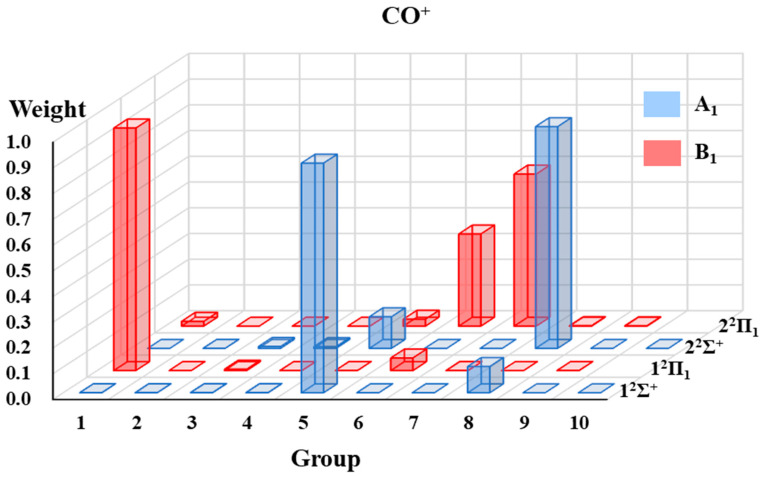
The weights of grouped VB structures for each state of CO^+^.

The VB structure weights gathered by groups for each state of the four radicals are displayed in Figure 2, Figure 3, Figure 4 and Figure 5. Since the ^2^Π state in the C_∞*v*_ point group is in two-fold degeneracy, each corresponding to the B_1_ and B_2_ states in the C_2v_ group, only the VB structural weights of the B_1_ states are illustrated.

In Figure 2, Figure 3, Figure 4 and Figure 5, the 1^2^Σ^+^ ground states of the four systems are all dominated by the structures in A_1_-G5 and followed by those in A_1_-G8. The A_1_-G5 corresponds to VB structures with an *σ* bond between atom A and B and one single electron in atom A and a lone pair in atom B, together with two-electron π-bonding in both the *x* and *y* directions respectively (see Figure 1). Although the VB structures in A_1_-G5 dominate the 1^2^Σ^+^ ground states, the weights of these structures for BO, CO^+^ and CN systems are distinguishable and descending as 0.94, 0.89, and 0.72. The decrease in the weights is in accordance with the decreasing electronegativity difference between the two bonded atoms of BO, CO^+^ and CN. For C_2_H, the weight of A_1_-G5 in the ground state is 0.91, which is between that of BO and CO^+^.

These four systems have the same first excited state 1^2^Π, which corresponds to two degenerate states 1^2^Π_1_ and 1^2^Π_2_. The dominating VB structures change from A_1_-G5 and A_1_-G8 in the 1^2^Σ^+^ state to the B_1_-G1 and B_1_-G6 in the 1^2^Π state. The weights of VB structures in B_1_-G1 exceed 0.9 for the 1^2^Π_1_ state of all the tested systems, while those in B_1_-G6 only have a small contribution. Therefore, the 1^2^Π_1_ state is mainly characterized as the excitation of one electron in the π orbital to the orbital with a single electron in atom A.

Although the four systems are isoelectronic and have some common features, some distinctions of the excited states could be captured. We will take the 2^2^Σ^+^ states of these four isoelectronic systems as examples. For C_2_H, although the weight of VB structures in A_1_-G5 decreases and that in A_1_-G8 increases for higher-lying states from 1^2^Σ^+^ to 2^2^Σ^+^, the VB structures in A_1_-G5 still have the largest contribution for the 2^2^Σ^+^ state. However, on the contrary to the C_2_H case, the VB structures in A_1_-G8 dominate the 2^2^Σ^+^ state of CN, CO^+^ and BO instead of A_1_-G5. As displayed in Figure 1, VB structures in A_1_-G8 are generated from A_1_-G5 by moving one electron from the lone pair of atom B to atom A. It is interesting to find that there exists another 1^2^Σ^−^ state between 2^2^Σ^+^ and 2^2^Π for C_2_H. For 1^2^Σ^−^ state, the main structures are A_2_-G5 and A_2_-G6 in Figure 1. For the 2^2^Π state, there also exists a difference in the primary structure groups between C_2_H and CN, CO^+^ and BO. The B_1_-G1 group dominates the wave function of the 2^2^Π state of C_2_H, while the main contributors for the 2^2^Π state of CN, CO^+^, and BO are B_1_-G6 and B_1_-G7. These special behaviors of 1^2^Σ^−^ and 2^2^Π states of C_2_H possibly arise from the appreciable Rydberg characters in such excited states, which we have mentioned above.

### 2.2. The PEC Study Along the Path with Avoided Crossing

**Figure 6:**
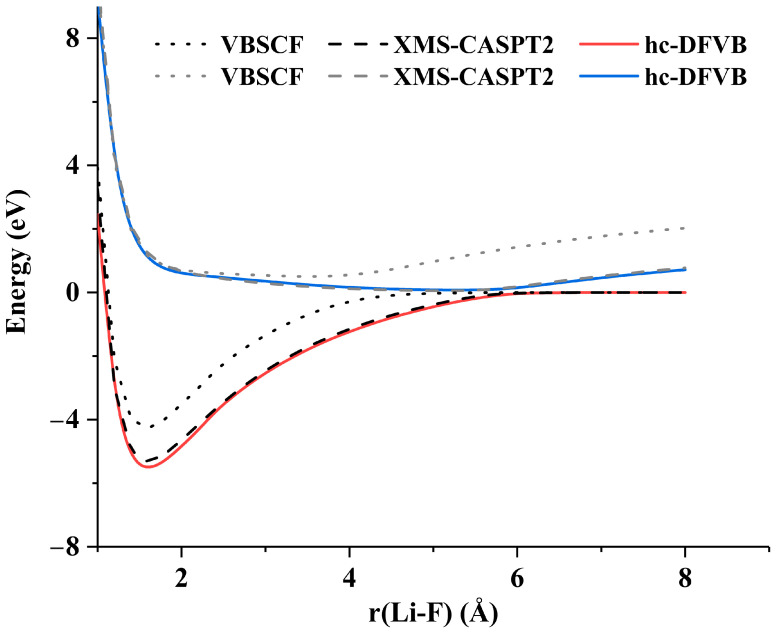
The PECs of the two lowest states of LiF calculated by XMS-CASPT2, VBSCF with HAOs, and hc-DFVB with HAOs and B3LYP functional.

**Figure 7:**
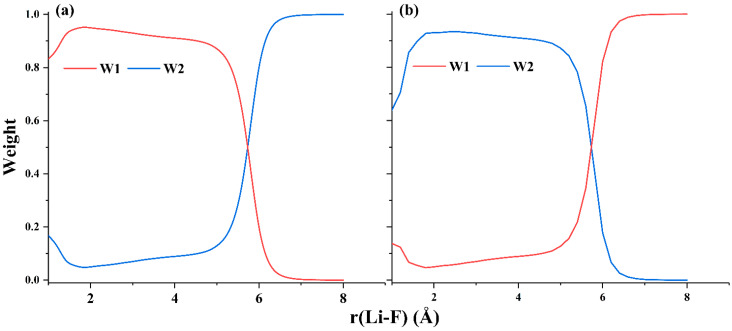
The weights of the two dominant VB structures of (**a**) the ground state and (**b**) the first excited state of LiF calculated by hc-DFVB with HAOs and B3LYP functional.

The PECs along paths with avoided crossings calculated by hc-DFVB for two systems, including LiF and the spiro cation are presented in Figure 6 and Figure 7, and are compared with those obtained with VBSCF and XMS-CASPT2.

**Figure 8:**
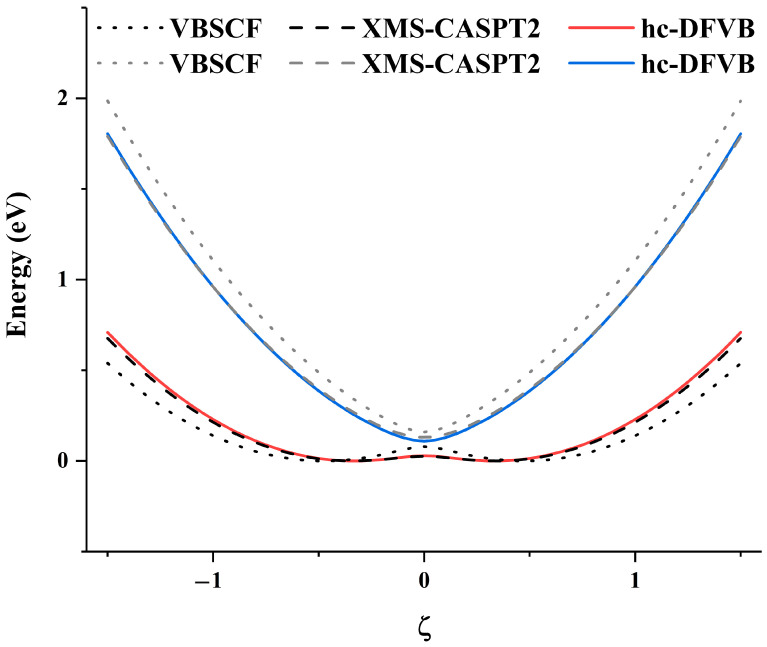
The PECs of the two lowest states of spiro cation calculated by XMS-CASPT2, VBSCF with block localized HAOs, and hc-DFVB with block localized HAOs and B3LYP functional.

The ground and first excited state PECs of LiF have been widely studied [45,46,48,49,50,51,52,53,54,55,56]. These two low-lying states both exhibit A_1_ irreducible representation in C_2v_ point group. The ground state at equilibrium bond length is dominated by the ionic structure Li^+^F^−^ and the first excited state is dominated by the covalent structure at equilibrium bond length. Figure 6 shows that hc-DFVB with HAOs and B3LYP functional gives the correct topology of the PECs of the two states; the PECs obtained with hc-DFVB are greatly improved over those obtained with VBSCF and are quite close to those obtained with XMS-CASPT2. Compared with VBSCF, which gives the minimum separation of 0.79 eV at 4.2 Å, both hc-DFVB and XMS-CASPT2 give much better descriptions at the avoided crossing region; the minimum separation between the two states at the avoided crossing region is 0.18 eV at 5.8 Å Li-F bond length for hc-DFVB, while XMS-CASPT2 gives the minimum separation of 0.18 eV at the 5.9 Å Li-F bond length. A better result, with a minimum separation of 0.09 eV at a 6.5 Å Li-F bond length, is obtained using our recently proposed hybrid DFVB method [35]. However, the exact bond length with the minimum separation is around 7.4 Å, which is significantly longer than those obtained by the aforementioned methods. This is because we only use two active orbitals in the calculation, one is the 2s orbital of Li and the other one is the 2p_z_ orbital of F. The distance will be closer to the accurate bond length when more active orbitals are included. Figure 8 displays the dominant VB structures along the dissociation of LiF, where W1 and W2 denote the ionic structure Li^+^F^−^ and the covalent structure Li-F, respectively. As shown in Figure 8, the dominant VB structure of the ground state around the equilibrium bond length is W1 and that at the dissociation limit is W2. While W2 is the dominant VB structure around the equilibrium bond length and W1 dominates at the dissociation limit for the first excited state. Figure 8 also shows that there is an interchange of the dominant structures along the L-F bond dissociation around 5.8 Å where the avoided crossing occurs.

**Figure 9:**
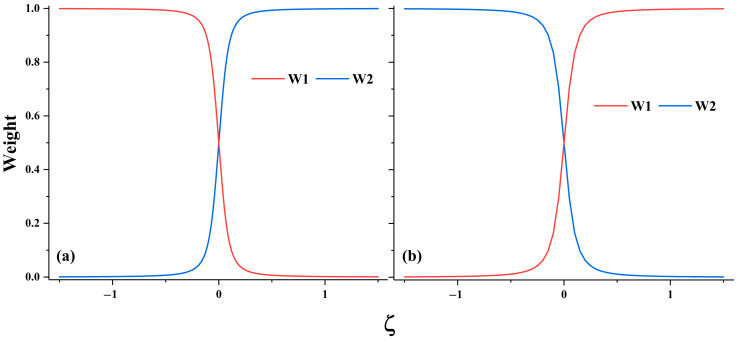
The weights of the two dominant VB structures of (**a**) the ground states and (**b**) the first excited state of spiro cation calculated by hc-DFVB with block localized HAOs and B3LYP functional.

The PECs of the first two low-lying states of the spiro cation are studied in Figure 8. The structure of the spiro cation can be found in Figure 1 in ref. [57]. The spiro cation is a mixed-valence compound that can be viewed as constructed by two subsystems (denoted as left and right subsystems) by removing one electron from the system. The two subsystems have the same structure in the neutral spiro molecule, however, the removal of one electron is mainly localized on either the left or right subsystem, resulting in a slight difference of the two subsystems at equilibrium geometry for the spiro cation. The equilibrium geometry when the positive charge localized on the left subsystem is denoted as geometry A ($Q_{\gamma }^{A}$), and the equilibrium geometry with the positive charge localized on the right subsystem is denoted as geometry B ($Q_{\gamma }^{B}$). As in ref. [57], the PECs studied are along the reaction path from geometry A to B with linear synchronous transit method as

(1) $$Q_{\gamma }\left(\xi \right)=\left(\frac{1}{2}-\xi \right)Q_{\gamma }^{A}+\left(\frac{1}{2}+\xi \right)Q_{\gamma }^{B},\;\gamma =1,2,\ldots ,3N_{\mathrm{atoms}}$$

where $Q_{\gamma }$ is the Cartesian coordinates of $N_{\mathrm{atoms}}$ atoms, and ξ is a parameter connecting geometries A and B ranging from −1.5 to 1.5. Specifically, ξ=−0.5, 0 and 0.5 correspond to the equilibrium geometry $Q_{\gamma }^{A}$, the transition state between $Q_{\gamma }^{A}$ and $Q_{\gamma }^{B}$, and the equilibrium geometry $Q_{\gamma }^{B}$, respectively. Figure 8 shows the PECs calculated by hc-DFVB using block localized HAOs and B3LYP functional. VBSCF, hc-DFVB and XMS-CASPT2 all give two local minimums of the ground state when ξ=±0.35, and the minimum deviation of 0.08 eV when ξ=0 for both VBSCF and hc-DFVB, and 0.10 eV for XMS-CASPT2. In Figure 9, the weights of the dominant VB structures of the two states along the reaction path are plotted, and W1 and W2 denote the VB structure with the positive charge localized on the left and right subsystems of the spiro cation, respectively. The dominant VB structure of the ground state is W1 when ξ<0 and W2 when ξ>0, while the dominant VB structure of the first excited state is W2 when ξ<0 and W1 when ξ>0. There is an interchange of the dominant structure at ξ=0, where the avoid crossing occurs. This can be interpreted as the positive charge transfer from the subsystem on the left to that on the right. 

## 3. Methodology

VBSCF is the fundamental method of VB theory. In VBSCF, a many-electron wave function is expressed as a linear combination of Heitler−London−Slater−Pauling (HLSP) functions {Φ*_K_*} as [58]:

(2) $$\Psi =\sum_{K}C_{K}\Phi_{K}$$

where Φ*_K_* is an HLSP function and corresponds to a specific VB structure and *C_K_* is the corresponding coefficient.

A VB structure Φ*_K_*can be expressed as:

(3) $$\Phi_{K}=\hat{A}\Omega_{K}\Theta_{K}$$

where $\hat{A}$ is the antisymmetrization operator, Ω*_K_* is a direct product of spatial orbitals $\left\{\phi_{K_{i}}\right\}$ occupied in the VB structure Φ*_K_*

(4) $$\Omega_{K}=\phi_{K_{1}}\left(1\right)\phi_{K_{2}}\left(2\right)\cdots \phi_{K_{N}}\left(N\right)$$

and $\Theta_{K}$ is a spin-paired eigenfunction defined as:

(5) $$\Theta_{K}=\prod_{\left(ij\right)}2^{-1/2}\left[\alpha \left(i\right)\beta \left(j\right)-\beta \left(i\right)\alpha \left(j\right)\right]\prod_{k}\alpha \left(k\right)$$

where (*ij*) runs over all covalent bonds and *k* runs over all unpaired electrons.

The electronic energy and structure coefficients can be obtained by solving the generalized secular equation:

(6) HC=EMC

where **H**, **M**, and **C** are the VBSCF Hamiltonian, overlap, and coefficient matrices, respectively.

The weights of VB structures can be used to explain the importance of each structure. One of the definitions for VB structure weight is provided by the Coulson–Chirgwin formula [59]:

(7) $$W_{K}=\sum_{L}C_{K}C_{L}M_{KL}$$

Negative weights might be obtained by using the above formula due to the large overlap between VB structures, and they can be avoided by using the Löwdin weight formula [60]:

(8) $$W_{K}={\left(\sum_{L}C_{L}S_{KL}^{1/2}\right)}^{2}$$

The hc-DFVB method accounts for the dynamic correlation by adding a correction term for each matrix element in VBSCF Hamiltonian, namely:

(9) $$H_{KL}^{\mathrm{hc}-\mathrm{DFVB}}=H_{KL}^{\mathrm{VBSCF}}+H_{KL}^{\mathrm{corr}}$$

The correction matrix element $H_{KL}^{\mathrm{corr}}$ can be calculated according to the contribution of each determinant pair as:

(10) $$H_{KL}^{\mathrm{corr}}=\sum_{\kappa \lambda }d_{\kappa K}d_{\lambda L}H_{\kappa \lambda }^{\mathrm{corr}}$$

where *d_κK_* is the expansion coefficient of determinant *D_κ_* in VB structure Φ*_K._* The diagonal contribution $H_{\kappa \kappa }^{\mathrm{corr}}$ is defined as:

(11) $$H_{\kappa \kappa }^{\mathrm{corr}}=E_{C}[\rho_{\kappa }]+(1-\alpha )(E_{X}[\rho_{\kappa }]-K_{\kappa })$$

where *ρ_κ_* is the electronic density of determinant *D_κ_*, while $E_{C}[\rho_{\kappa }]$, $E_{X}[\rho_{\kappa }]$ are the correlation and exchange energies computed from the DFT functional, the parameter *α* is the ratio of exact exchange energies used in the corresponding hybrid DFT functional, and $K_{\kappa }$ is the exact exchange obtained from determinant *D_κ_*, and is defined as:

(12) $$K_{\kappa }=\sum_{\mu \nu \sigma \lambda }P_{\mu \nu }^{\kappa }P_{\sigma \lambda }^{\kappa }g_{\nu \lambda ,\sigma \mu }$$

where **P***^κ^* is the density matrix of VB determinant *D_κ_* and *g_νλ_*_,*σμ*_ is the two-electron integral.

The off-diagonal contribution $H_{\kappa \lambda }^{\mathrm{corr}}$, where κ≠λ, can be approximated by either

(13) $$H_{\kappa \lambda }^{\mathrm{corr}}=\frac{H_{\kappa \lambda }^{\mathrm{VBSCF}}}{H_{\kappa \kappa }^{\mathrm{VBSCF}}+H_{\lambda \lambda }^{\mathrm{VBSCF}}}(H_{\kappa \kappa }^{\mathrm{corr}}+H_{\lambda \lambda }^{\mathrm{corr}})$$

or

(14) $$H_{\kappa \lambda }^{\mathrm{corr}}=\frac{1}{2}S_{\kappa \lambda }(H_{\kappa \kappa }^{\mathrm{corr}}+H_{\lambda \lambda }^{\mathrm{corr}})$$

where $H_{\kappa \lambda }^{\mathrm{VBSCF}}$ is the VBSCF Hamiltonian matrix element between determinants *D_κ_*and *D_λ_*, and *S_κλ_* is their overlap matrix element. In the original hc-DFVB paper, Equation (13) is employed, while the application of the simpler Equation (14) is proved to give similar results compared with those obtained with Equation (13). Therefore, all the hc-DFVB calculations in this paper will be carried out using Equation (14) to avoid the calculation of $H_{\kappa \lambda }^{\mathrm{VBSCF}}$ between nonorthogonal determinants.

## 4. Computational Details

All the VB calculations are performed in the Xiamen Valence Bond (XMVB) package v4.0 [61,62]. The MS-CASPT2 and XMS-CASPT2 calculations are performed in *OpenMolcas* v21.10 [63]. Imaginary shift [64] of 0.2 hartrees is applied for both MS-CASPT2 and XMS-CASPT2 calculations and the default IPEA shift [65] of 0.25 hartrees is applied in the MS-CASPT2 calculations, while no IPEA shift is applied in the XMS-CASPT2 calculations. The MRCISD+Q calculations are performed in the MOLPRO program (version 2010.1) [66,67].

The low-lying excited states of four small doublet radicals (C_2_H, CN, BO, CO^+^) are studied in Section 2.1 using the aug-cc-pVTZ basis set. The purely localized orbitals centered on an atom or a fragment (referred to as hybrid atomic orbitals, HAOs) are used for these four radicals. Three DFT functionals, namely B3LYP [68], BLYP [69,70], and PBE [71], are applied in hc-DFVB calculations. Table 5 lists the number of states for each state symmetry considered in the state-averaged VBSCF (SA-VBSCF) calculations, active spaces for SA-VBSCF, and state-averaged CASSCF (SA-CASSCF) calculations and references from which the molecular geometries are taken. All the SA-VBSCF calculations are performed for states with the same symmetry. The weights of VB structures classified according to point group symmetry (see Section 2.1.1 for details) are presented. Since the weights calculated with VBSCF and different functionals by hc-DFVB do not have much difference, only the hc-DFVB weights with B3LYP functional are presented in Figure 2, Figure 3, Figure 4 and Figure 5. The detailed results of VB structural weights are presented in Supplementary Materials.

The PECs along the path with avoided crossing of LiF and the mixed-valence spiro cation are studied in Section 2.2, Table 6 presents the basis sets, numbers of states in the SA calculation and active spaces for SA-VBSCF and SA-CASSCF calculations, and the orbital types and the characters of each active orbitals for the tested systems. Since the hc-DFVB method with different functionals gives similar overall shapes of the PECs, only the hc-DFVB PECs with B3LYP functional are plotted in Figure 6 and Figure 7, while the PECs with other functionals are presented in Supplementary Materials.

## 5. Conclusions

In this work, the electronic structures of the low-lying excited states of four isoelectronic systems (C_2_H, CN, CO^+^, BO), and the PECs for LiF and a mixed-valence spiro cation are studied with the hc-DFVB method. The hc-DFVB method is a valence-bond-based multi-reference density functional theory that accounts for dynamic correlation by adding correction terms to the VBSCF Hamiltonian matrix elements, effectively handling strong correlation effects and state interactions.

In the calculations of isoelectronic systems, computation results show that the hc-DFVB method can achieve much better excitation energies compared to VBSCF when the HAOs are used. Furthermore, the correct ordering of each excited state can always be predicted by hc-DFVB, while there exists some inverse ordering of states in VBSCF. Instead of selecting the VB structures carefully, we divided the VB structures into different groups by applying the point group symmetry. For each state, we can easily find its important structure contribution and obtain a clear bonding picture from the weight distribution of structure groups. By comparing the weight distribution variation between the ground state and excited states, the transition feature of each excited state can be easily understood. Although the four systems are isoelectronic, there are some quantitative differences in the weight of dominant VB structures due to the electronegativity difference between the two bonding atoms. For the high excited states, in particular, the structure weights usually vary a lot among the studying systems.

In the calculations of LiF and the mixed-valence spiro cation, hc-DFVB is shown to give the correct topology of the PECs near the strongly state-interaction regions. The weight analysis also indicates the nature of the state interaction in the avoid crossing region. Therefore, hc-DFVB is shown to be an accurate and efficient multi-state method.

Overall, hc-DFVB is a multi-state method in which static and dynamic correlation is considered in a balanced way and is promising to be applied to the study of excited states in the perspective of valence bond theory.

## Figures and Tables

**Table 1:** The vertical excitation energies obtained with MRCISD+Q and the deviation of vertical excitation energies from MRCISD+Q calculated by various methods of C_2_H. The colored cells indicate the magnitude of the errors, with colors transitioning from green (small errors) to red (large errors). All the values are in eV.

State	MRCISD+Q	MS-CASPT2	VBSCF	hc-DFVB		
				B3LYP	BLYP	PBE
1^2^Π_1_/1^2^Π_2_	0.69	0.15	0.39	0.29	0.34	0.51
2^2^Σ^+^	6.94	0.11	1.20	0.91	0.82	0.82
1^2^Σ^−^	7.39	0.15	1.46	1.10	1.03	1.14
2^2^Π_1_/2^2^Π_2_	7.56	0.26	1.10	0.96	0.98	1.15
	MUD	0.17	1.04	0.82	0.79	0.91

**Table 2:** The vertical excitation energies obtained with MRCISD+Q and the deviation of vertical excitation energies from MRCISD+Q calculated by various methods of CN. The colored cells indicate the magnitude of the errors, with colors transitioning from green (small errors) to red (large errors). All the values are in eV.

State	MRCISD+Q	MS-CASPT2	VBSCF	hc-DFVB		
				B3LYP	BLYP	PBE
1^2^Π_1_/1^2^Π_2_	1.32	0.12	0.33	0.32	0.38	0.53
2^2^Σ^+^	3.17	0.03	0.34	0.31	0.31	0.34
2^2^Π_1_/2^2^Π_2_	7.83	0.03	0.65	0.24	0.10	0.06
	MUD	0.06	0.44	0.29	0.26	0.31

**Table 3:** The vertical excitation energies obtained with MRCISD+Q and the deviation of vertical excitation energies from MRCISD+Q calculated by various methods of BO. The colored cells indicate the magnitude of the errors, with colors transitioning from green (small errors) to red (large errors). All the values are in eV.

State	MRCISD+Q	MS-CASPT2	VBSCF	hc-DFVB		
				B3LYP	BLYP	PBE
1^2^Π_1_/1^2^Π_2_	3.58	−0.10	−0.01	−0.20	−0.18	−0.07
2^2^Σ^+^	5.60	−0.05	0.19	0.10	0.09	0.19
2^2^Π_1_/2^2^Π_2_	7.19	0.09	0.73	0.27	0.15	0.16
	MUD	0.08	0.31	0.19	0.14	0.14

**Table 4:** The vertical excitation energies obtained with MRCISD+Q and the deviation of vertical excitation energies from MRCISD+Q calculated by various methods of CO^+^. The colored cells indicate the magnitude of the errors, with colors transitioning from green (small errors) to red (large errors). All the values are in eV.

State	MRCISD+Q	MS-CASPT2	VBSCF	hc-DFVB		
				B3LYP	BLYP	PBE
1^2^Π_1_/1^2^Π_2_	3.30	0.01	0.18	0.01	0.05	0.19
2^2^Σ^+^	5.80	0.01	0.34	0.14	0.11	0.21
2^2^Π_1_/2^2^Π_2_	9.27	−0.09	0.39	−0.13	−0.27	−0.26
	MUD	0.04	0.30	0.09	0.14	0.22

**Table 5:** The number of states in SA-VBSCF calculation (*N*_states_ for each state symmetry), number of active electrons (*n*), number of active orbitals (*m*), and references from which the molecular geometries are taken.

Molecule	(*n*, *m*)	State Symmetry (*N*_states_)	Reference
C_2_H	(7, 6)	^2^Π_1_(2), ^2^Π_2_(2), ^2^Σ^+^(2), ^2^Σ^−^(2)	[72]
CN	(7, 6)	^2^Π_1_(2), ^2^Π_2_(2), ^2^Σ^+^(2)	[73]
BO	(7, 6)	^2^Π_1_(2), ^2^Π_2_(2), ^2^Σ^+^(2)	[73]
CO^+^	(7, 6)	^2^Π_1_(2), ^2^Π_2_(2), ^2^Σ^+^(2)	[73]

**Table 6:** The basis set, numbers of states (*N*_states_) in the SA calculation, active electrons (*n*), active orbitals (*m*), the VB orbital types, and the characters of each active orbitals for LiF and the mixed-valence spiro cation systems. The details of active orbitals are shown in Supplementary Materials.

Molecule	Basis Set	*N*_states_	(*n*, *m*)	VB Orbital Type	Active Orbitals
LiF	aug-cc-pVTZ	2	(2, 2)	HAOs	2p_z_ of F, 2s of Li
Spiro cation	6–31G*	2	(7, 4)	block localized HAOs	Two π orbitals located on each subsystem

## Data Availability

Data are contained within the article and Supplementary Materials.

## Supplementary Materials

The following supporting information can be downloaded at: https://www.mdpi.com/article/10.3390/molecules30030489/s1, Figure S1: Active orbitals used in the calculation of LiF. The VB orbitals are plotted using isovalue 0.05, Figure S2: Active orbitals used in the calculation of the spiro cation. The VB orbitals are plotted using isovalue 0.03, Figure S3: The PECs of the two lowest states of LiF calculated by XMS-CASPT2, VBSCF with HAOs, and hc-DFVB with HAOs and BLYP functional, Figure S4: The PECs of the two lowest states of LiF calculated by XMS-CASPT2, VBSCF with HAOs, and hc-DFVB with HAOs and M06 functional, Figure S5: The PECs of the two lowest states of LiF calculated by XMS-CASPT2, VBSCF with HAOs, and hc-DFVB with HAOs and revTPSS functional, Figure S6: The PECs of the two lowest states of spiro cation calculated by XMS-CASPT2, VBSCF with block localized HAOs, and hc-DFVB with block localized HAOs and BLYP functional, Figure S7: The PECs of the two lowest states of spiro cation calculated by XMS-CASPT2, VBSCF with block localized HAOs, and hc-DFVB with block localized HAOs and M06 functional, Figure S8: The PECs of the two lowest states of spiro cation calculated by XMS-CASPT2, VBSCF with block localized HAOs, and hc-DFVB with block localized HAOs and revTPSS functional, Table S1: The number of VB structures for each group of C_2_H, CN, BO, and CO^+^, Table S2: The weights of grouped VB structures for each state of C_2_H, Table S3: The weights of grouped VB structures for each state of CN, Table S4: The weights of grouped VB structures for each state of BO, Table S5: The weights of grouped VB structures for each state of CO^+^, Table S6: The deviation of vertical excitation energies from MRCISD+Q calculated by U-TD-DFT and hc-DFVB of the C_2_H, CN and CO^+^ systems. The U-TD-DFT values are taken from the reference: Li, Z.; Liu, W. Critical Assessment of TD-DFT for Excited States of Open-Shell Systems: I. Doublet–Doublet Transitions. *J. Chem. Theory Comput.* **2016**, 12, 238-260, doi:10.1021/acs.jctc.5b01158 [74]. All the values are in eV.
