# Supplementary material for: A Density Functional Valence Bond Study on the Excited States
Source: Molecules. 2025 Jan 22;30(3):489. doi: 10.3390/molecules30030489 (PMC11820789; doi:10.3390/molecules30030489)
Supplement: Supplementary file 1 [file molecules-30-00489-s001.zip › molecules-3421500-supplementary.pdf]

SUPPORTING INFORMATION

A Density Functional Valence Bond Study on the Excited States

Xun Wu, Peikun Zheng, Tingzhen Chen, Chen Zhou *, Peifeng Su and Wei Wu *

State Key Laboratory of Physical Chemistry of Solid Surfaces, Fujian Provincial Key Laboratory of Theoretical and Computational Chemistry, Department of Chemistry, College of Chemistry and Chemical Engineering, Xiamen University, Xiamen 361005, China

* Correspondence: czhou1989@xmu.edu.cn (C.Z.); weiwu@xmu.edu.cn (W.W.)

Table S1. The number of VB structures for each group of C_2H, CN, BO, and CO^+.

	1	2	3	4	5	6	7	8	9	10	total
A_1	4	4	4	4	17	1	1	17	1	1	54
A_2	8	8	8	8	8	8	—	—	—	—	48
B_1	8	2	2	4	8	8	18	2	2	—	54
B_2	8	2	2	4	8	8	18	2	2	—	54

Table S2. The weights of grouped VB structures for each state of C_2H.

	$1^2\Sigma^+$		$1^2\Pi$		$2^2\Sigma^+$		$1^2\Sigma^-$		$2^2\Pi$	
	VBSCF	hc-DFVB (B3LYP)	VBSCF	hc-DFVB (B3LYP)	VBSCF	hc-DFVB (B3LYP)	VBSCF	hc-DFVB (B3LYP)	VBSCF	hc-DFVB (B3LYP)
1	0.000	0.000	0.984	0.979	0.000	0.000	0.000	0.000	0.968	0.957
2	0.000	0.000	0.000	0.000	0.000	0.000	0.001	0.001	0.000	0.000
3	0.000	0.001	0.003	0.004	0.000	0.000	0.036	0.037	0.003	0.004
4	0.000	0.001	0.000	0.000	0.000	0.000	0.030	0.030	0.000	0.001
5	0.919	0.911	0.001	0.002	0.774	0.783	0.510	0.513	0.002	0.002
6	0.000	0.000	0.010	0.013	0.000	0.000	0.424	0.419	0.012	0.017
7	0.000	0.000	0.001	0.001	0.000	0.000	—	—	0.001	0.001
8	0.080	0.087	0.000	0.000	0.225	0.216	—	—	0.000	0.000
9	0.000	0.000	0.000	0.000	0.000	0.000	—	—	0.014	0.018
10	0.000	0.000	—	—	0.000	0.000	—	—	—	—

Table S3. The weights of grouped VB structures for each state of CN.

	$1^2\Sigma^+$		$1^2\Pi$		$2^2\Sigma^+$		$2^2\Pi$	
	VBSCF	hc-DFVB (B3LYP)	VBSCF	hc-DFVB (B3LYP)	VBSCF	hc-DFVB (B3LYP)	VBSCF	hc-DFVB (B3LYP)
1	0.002	0.002	0.976	0.971	0.000	0.001	0.012	0.081

2	0.001	0.002	0.001	0.001	0.000	0.000	0.000	0.001
3	0.001	0.002	0.002	0.003	0.002	0.003	0.000	0.078
4	0.002	0.002	0.000	0.000	0.003	0.003	0.000	0.002
5	0.738	0.722	0.003	0.003	0.267	0.281	0.060	0.082
6	0.000	0.000	0.018	0.022	0.000	0.000	0.149	0.090
7	0.000	0.000	0.000	0.000	0.000	0.000	0.772	0.666
8	0.256	0.270	0.000	0.000	0.727	0.712	0.002	0.000
9	0.000	0.000	0.000	0.001	0.000	0.000	0.005	0.000
10	0.000	0.000	—	—	0.000	0.000	—	—

Table S4. The weights of grouped VB structures for each state of BO.

	$1^2\Sigma^+$		$1^2\Pi$		$2^2\Sigma^+$		$2^2\Pi$	
	VBSCF	hc-DFVB (B3LYP)	VBSCF	hc-DFVB (B3LYP)	VBSCF	hc-DFVB (B3LYP)	VBSCF	hc-DFVB (B3LYP)
1	0.000	0.001	0.937	0.918	0.000	0.000	0.000	0.000
2	0.001	0.001	0.000	0.000	0.000	0.000	0.000	0.000
3	0.001	0.001	0.004	0.005	0.005	0.007	0.005	0.007
4	0.001	0.001	0.000	0.000	0.004	0.005	0.004	0.005
5	0.945	0.942	0.000	0.000	0.068	0.070	0.068	0.070
6	0.000	0.000	0.057	0.075	0.000	0.000	0.000	0.000
7	0.000	0.000	0.001	0.002	0.000	0.000	0.000	0.000
8	0.053	0.056	0.000	0.000	0.922	0.918	0.922	0.918
9	0.000	0.000	0.000	0.000	0.000	0.000	0.000	0.000
10	0.000	0.000	—	—	0.000	0.000	—	—

Table S5. The weights of grouped VB structures for each state of CO^+.

	$1^2\Sigma^+$		$1^2\Pi$		$2^2\Sigma^+$		$2^2\Pi$	
	VBSCF	hc-DFVB (B3LYP)	VBSCF	hc-DFVB (B3LYP)	VBSCF	hc-DFVB (B3LYP)	VBSCF	hc-DFVB (B3LYP)
1	0.001	0.001	0.955	0.943	0.000	0.000	0.011	0.019
2	0.001	0.001	0.000	0.000	0.000	0.000	0.000	0.000
3	0.001	0.001	0.005	0.005	0.006	0.008	0.000	0.000
4	0.001	0.001	0.000	0.000	0.006	0.007	0.000	0.000
5	0.903	0.893	0.001	0.001	0.116	0.123	0.028	0.027
6	0.000	0.000	0.039	0.049	0.000	0.000	0.284	0.358
7	0.000	0.000	0.001	0.001	0.000	0.000	0.673	0.591
8	0.093	0.102	0.000	0.000	0.872	0.862	0.002	0.002
9	0.000	0.000	0.001	0.001	0.000	0.000	0.001	0.002
10	0.000	0.000	—	—	0.000	0.000	—	—

Table S6. The deviation of vertical excitation energies from MRCISD+Q calculated by U-TD-DFT and hc-DFVB of the C_2H, CN and CO^+ systems. The U-TD-DFT values are taken from the reference: Li, Z.; Liu, W. Critical Assessment of TD-DFT for Excited States of Open-Shell Systems: I. Doublet–Doublet Transitions. J. Chem. Theory Comput. 2016, 12, 238-260, doi:10.1021/acs.jctc.5b01158. All the values are in eV.

Molecule	State	U-TD-DFT		hc-DFVB	
		B3LYP	BLYP	B3LYP	BLYP
C_2H	$1^2\Pi_1/1^2\Pi_2$	-0.10	-0.29	0.29	0.34
	$2^2\Sigma^+$	-1.68	-1.51	0.91	0.82
	$1^2\Sigma^-$	-0.58	-0.49	1.10	1.03
	$2^2\Pi_1/2^2\Pi_2$	-	-	0.96	0.98
	MUD	-	-	0.82	0.79
CN	$1^2\Pi_1/1^2\Pi_2$	-0.25	-0.06	0.32	0.38
	$2^2\Sigma^+$	-0.07	0.09	0.31	0.31
	$2^2\Pi_1/2^2\Pi_2$	0.42	0.29	0.24	0.10
	MUD	0.25	0.15	0.29	0.26
CO^+	$1^2\Pi_1/1^2\Pi_2$	-0.01	-0.25	0.01	0.05
	$2^2\Sigma^+$	-0.23	-0.75	0.14	0.11
	$2^2\Pi_1/2^2\Pi_2$	-0.07	-0.19	-0.13	-0.27
	MUD	0.10	0.40	0.09	0.14

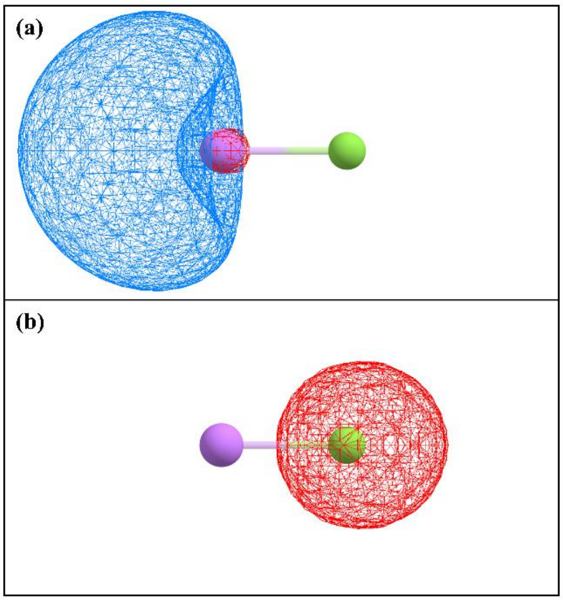

Figure S1. Active orbitals used in the calculation of LiF. The VB orbitals are plotted using isovalue 0.05.

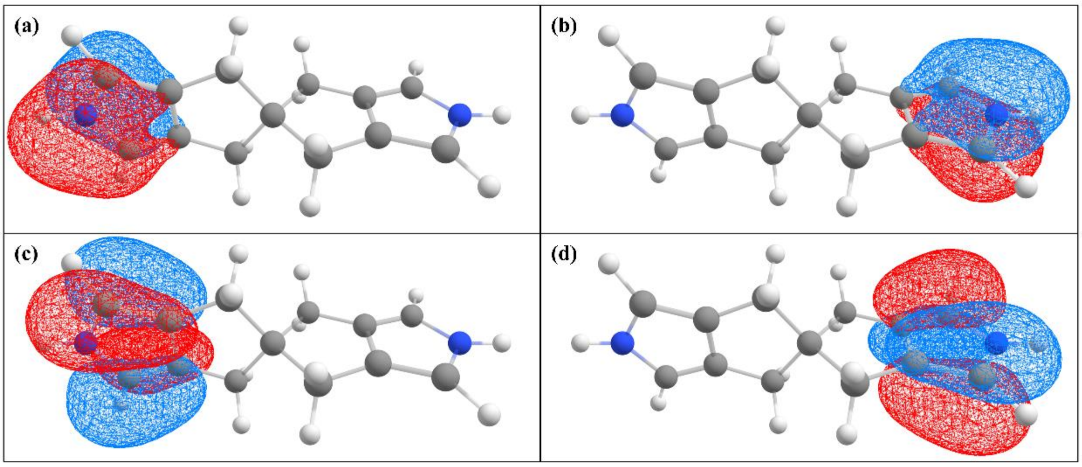

Figure S2. Active orbitals used in the calculation of the spiro cation. The VB orbitals are plotted using isovalue 0.03.

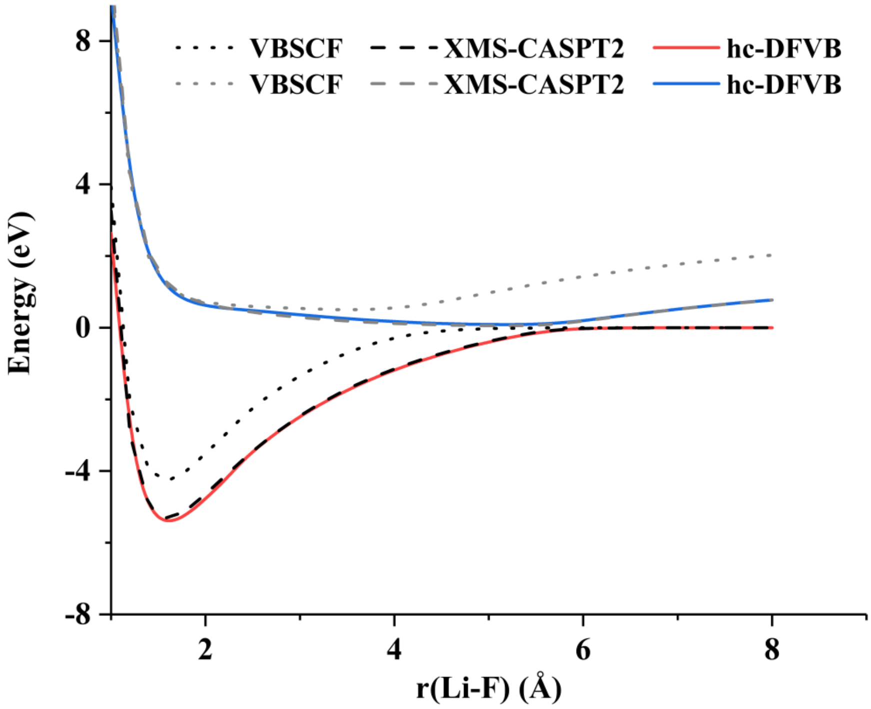

Figure S3. The PECs of the two lowest states of LiF calculated by XMS-CASPT2, VBSCF with HAOs, and hc-DFVB with HAOs and BLYP functional.

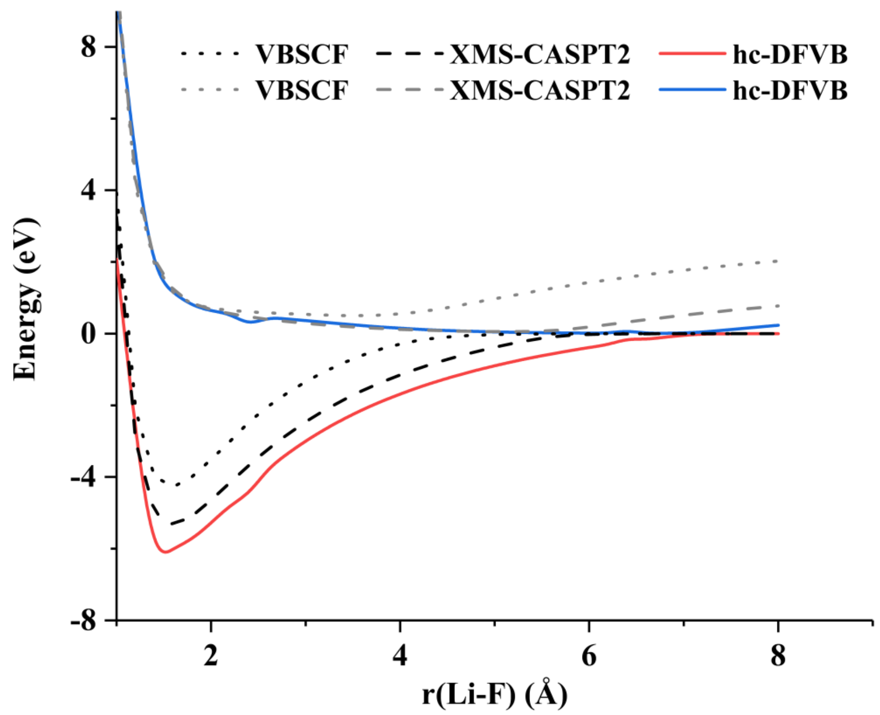

Figure S4. The PECs of the two lowest states of LiF calculated by XMS-CASPT2, VBSCF with HAOs, and hc-DFVB with HAOs and M06 functional.

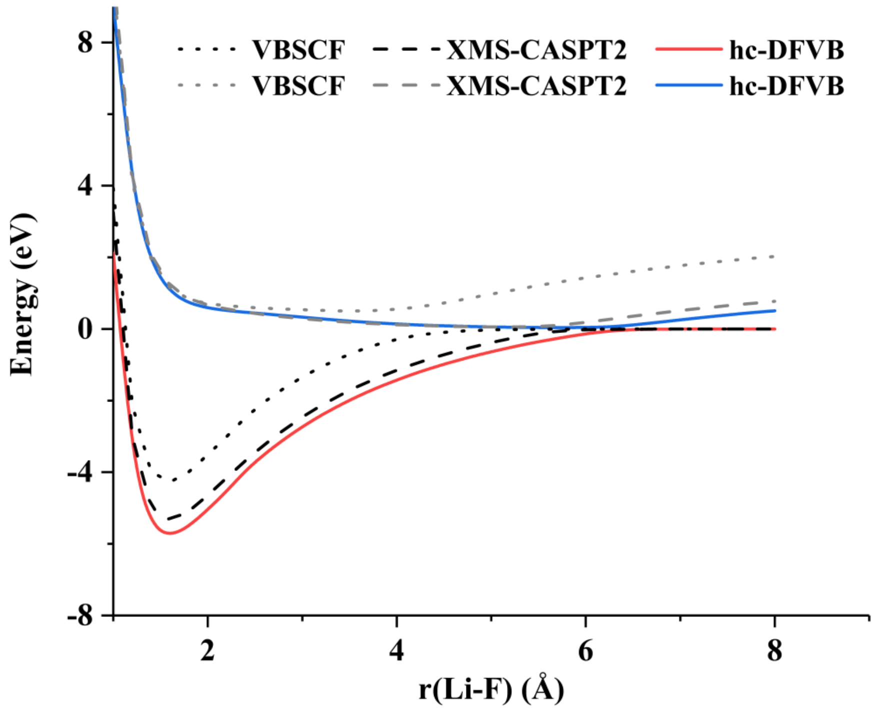

Figure S5. The PECs of the two lowest states of LiF calculated by XMS-CASPT2, VBSCF with HAOs, and hc-DFVB with HAOs and revTPSS functional.

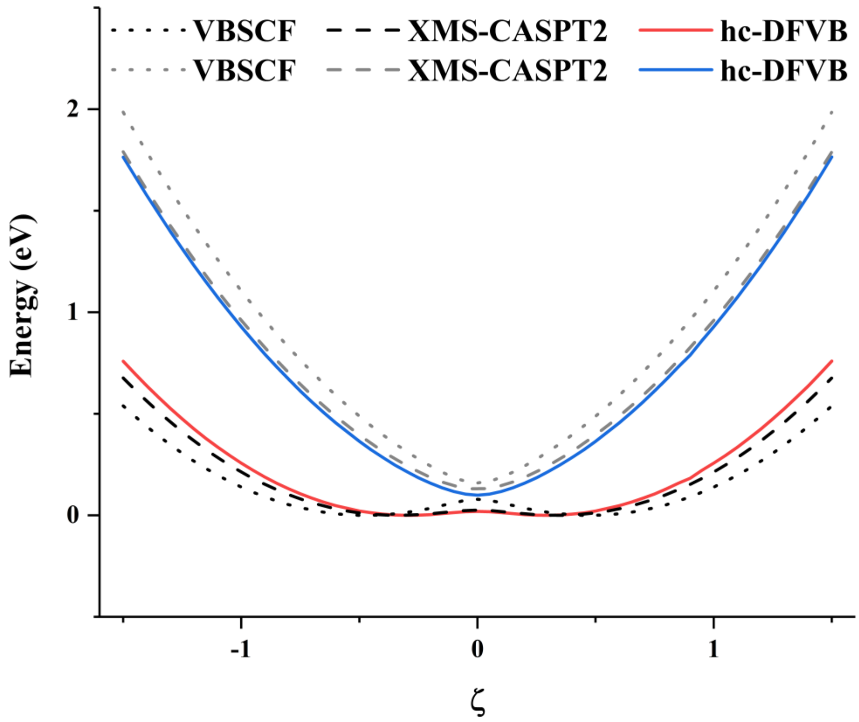

Figure S6. The PECs of the two lowest states of spiro cation calculated by XMS-CASPT2, VBSCF with block localized HAOs, and hc-DFVB with block localized HAOs and BLYP functional.

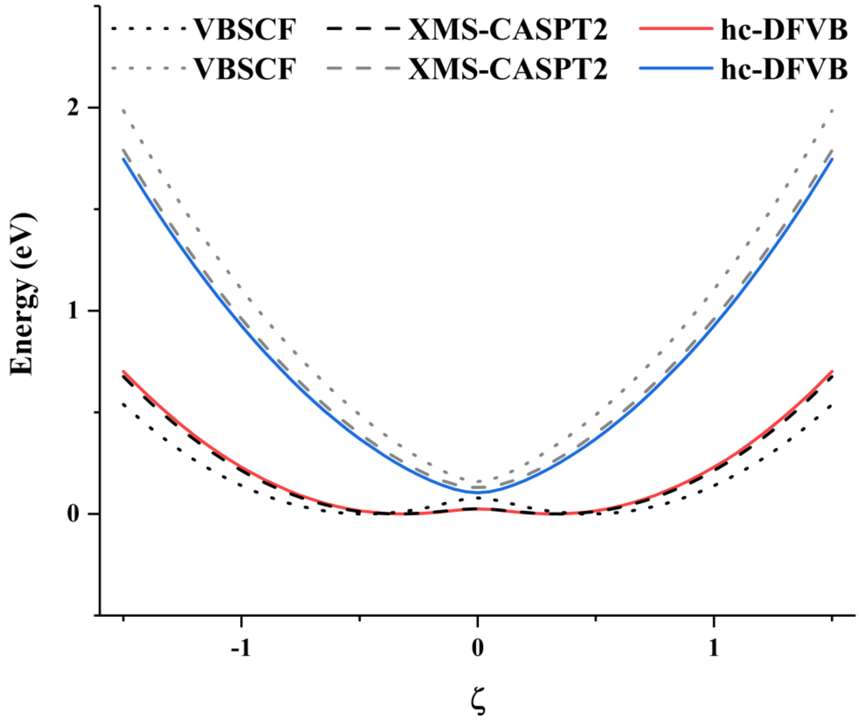

Figure S7. The PECs of the two lowest states of spiro cation calculated by XMS-CASPT2, VBSCF with block localized HAOs, and hc-DFVB with block localized HAOs and M06 functional.

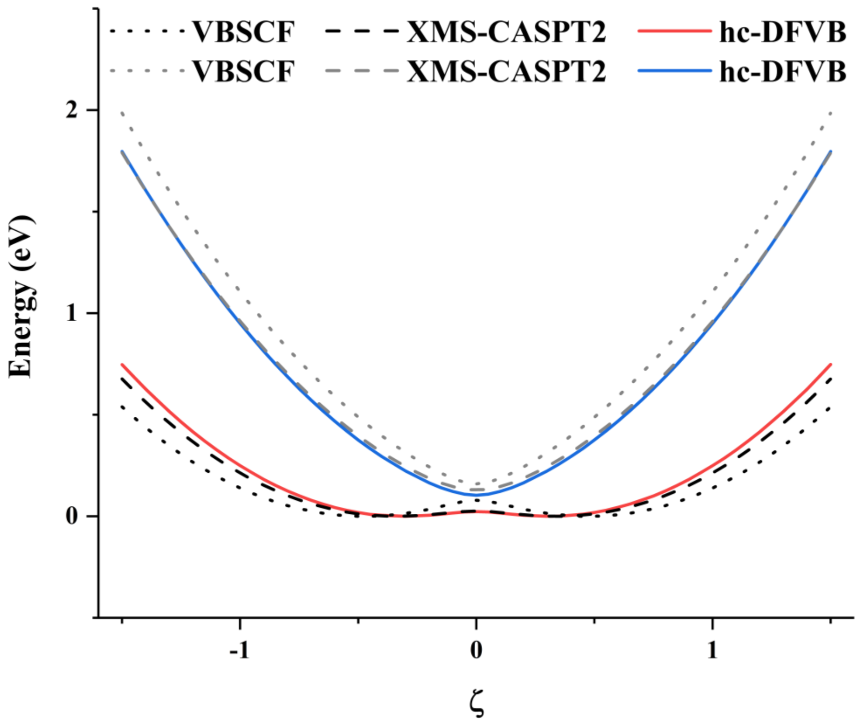

Figure S8. The PECs of the two lowest states of spiro cation calculated by XMS-CASPT2, VBSCF with block localized HAOs, and hc-DFVB with block localized HAOs and revTPSS functional.
